# Improving judgment accuracy by sequential adjustment

**DOI:** 10.3758/s13423-019-01696-5

**Published:** 2020-01-02

**Authors:** Shenghua Luan, Lael J. Schooler, Jolene H. Tan

**Affiliations:** 1 CAS Key Laboratory of Behavioral Sciences, Chinese Academy of Sciences, Lincui Road 16, Chaoyang District, Beijing, 100101 China; 2 Department of Psychology, Syracuse University, Syracuse, NY USA; 3 Max Planck Research Group Naturalistic Social Cognition, Max Planck Institute for Human Development, Berlin, Germany

**Keywords:** Judgment, Sequential processing, Heuristics, Decision analysis, Ecological rationality

## Abstract

Judging an object’s value based on relevant cues can be challenging. We propose a simple method to improve judgment accuracy: Instead of estimating a value after seeing all available cues simultaneously, individuals view cues sequentially, one after another, making and adjusting their estimate at each step. The sequential procedure may alleviate computational difficulties in cue integration, leading to higher judgment accuracy. We tested this hypothesis in two real-world tasks in which participants judged either the price of diamonds or the fuel economy of cars. Two studies with professional jewelers and car salespeople show that most participants indeed judged more accurately with a sequential than with a simultaneous procedure. Another two studies with college students further support this finding and show additionally that the sequential procedure could raise the judgment accuracy of inexperienced students to the same level as that of professionals judging with the simultaneous procedure.

**Electronic supplementary material:**

The online version of this article (10.3758/s13423-019-01696-5) contains supplementary material, which is available to authorized users.

## Introduction

People routinely judge an object’s value. In most cases, because the value is not directly observable (e.g., the monetary worth of an antique), it has to be estimated through relevant cues or features (e.g., age and condition; Brunswik, 1952). Previous studies have shown that people often have trouble integrating information from multiple cues to form value judgments; as a result, the accuracy of their judgments usually falls well below the ideal level (e.g., Hammond & Stewart, 2001; Karelaia & Hogarth, 2008). Consider a real-world example that demonstrates how challenging cue integration can be.

In the consumer world, diamond price is known to depend on the so-called 4Cs: carat, color, clarity, and cut. What, then, is the expected price of a diamond given its values on these cues? To find out, we ran regressions on 37,815 diamonds. The *R*^2^ of the regression is .98 with all four cues as predictors for price and is .94 with just carat and color. In the latter case, the regression equation is

$$ \mathrm{Log}\left(\mathrm{price}\right)=3.76+2.05\times \mathrm{Log}\left(\mathrm{carat}\right)+0.37\times \mathrm{Log}\left(\mathrm{color}\right). $$

If a diamond weighs 0.61 carats and has a color grade of “F” or 5 on a numerical scale,

1 its expected price is then 10^3.76+2.05×Log(0.61)+0.37×Log(5)^ =$3,789.

Regression is a popular statistical tool the core mechanism of which is weighting-and-adding: Cues are weighted according to their importance and their weighted values added to arrive at an overall estimate. Cognitive models with the same mechanism have been proposed in many areas of psychology, including judgment. The general finding is that such models can describe well how people integrate information from multiple cues (see a review in Karelaia & Hogarth, 2008). Nonetheless, anyone who tries to weight and add the carat and color cues to estimate diamond price needs to deal with the following issues.

First, because of the nonlinear relationships (i.e., power) between price and the cues and the multiplicative effects of the cues on price (i.e., a better color will increase price more on larger diamonds than smaller ones), one needs to transform the variables’ original values to something akin to the logarithmic values to make accurate judgments. Second, one needs to apply a set of parameters, including cue weights and the linear constant, when combining cue information. Estimating these parameters, however, can be difficult without extensive learning (e.g., Hammond & Stewart, 2001). One reason is that cues are typically measured in different scales; to figure out how to align and compare them side by side requires both experience and effort. Third, one needs to perform multiple mathematical operations before getting an estimate. Without the help of some external device, these operations can be mentally taxing and error prone (e.g., Payne, Bettman, & Johnson, 1993). The errors people commit while trying to resolve these issues explain why their judgments are usually less accurate than what they could be with statistical regression.

**Fig. 1:**
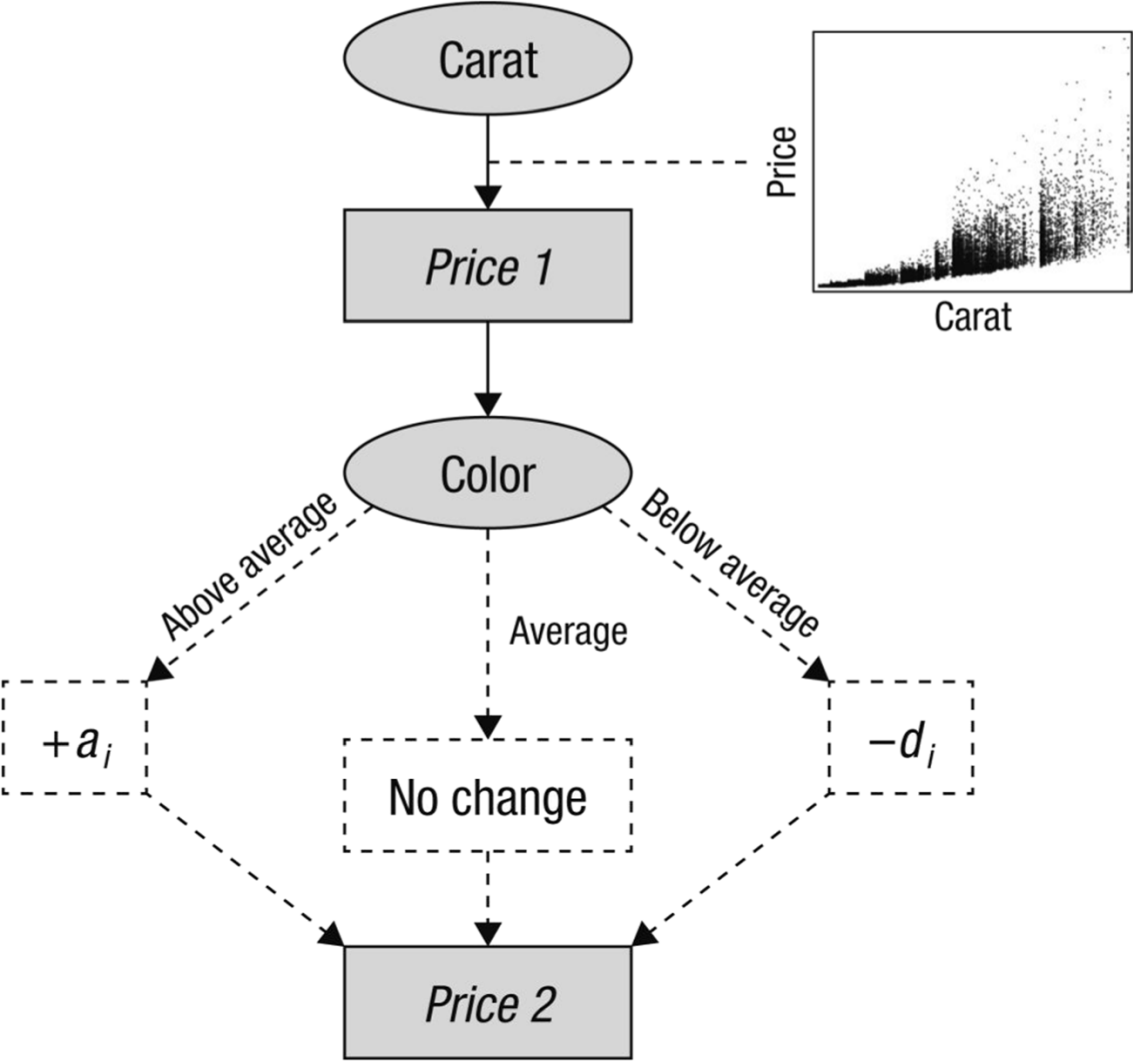
Illustration of the sequential adjustment process using the example of estimating diamond price based on first the carat and then the color cues. The graph in the figure depicts the relationship between carat and price in the diamond data set collected in Study 2

To assess judgment accuracy, researchers typically present all cues at once and ask participants to provide an estimate afterward. This “simultaneous” procedure, in terms of cue presentation, promotes a weighting-and-adding approach to information integration, because of people’s strong tendency to weight-and-add in the absence of the need to search for and update information (e.g., Rieskamp & Otto, 2006). Meanwhile, in judgment studies where accuracy is difficult to assess (e.g., personal impressions), a “sequential” procedure, in which cues are presented one by one and participants provide an estimate at each step, has also been applied. This procedure promotes a “sequential adjustment” approach to information integration (e.g., Hogarth & Einhorn, 1992; Juslin, Karlsson, & Olsson, 2008). An example of this approach applied to judging diamond price is illustrated in Fig. 1.

The first cue encountered is carat, the most conspicuous property of a diamond. A person makes an estimate, *Price 1*, based on their understanding of the relationship between carat and price. After that, they are given the color cue and can revise the initial estimate. On the basis of previous research (Hogarth & Einhorn, 1992), we assume that they would add a value of *a*_*i*_ to *Price 1* if the color is above average, deduct *d*_*i*_ if it is below average, and make no change if it is average. Finally, if more cues are available, the adjustment process will continue in a similar fashion; if not, *Price 2* will be the final estimate.

To estimate the price of a 0.61-carat diamond with an above-average color of “F,” a person might first give a ballpark estimate of $3,500 based on the carat cue alone and then add another $300 after the color is revealed. Errors will occur if the carat-to-price function is inaccurate and an inappropriate adjustment value is applied. However, in comparison to weighting-and-adding, this approach can reduce the difficulty of cue integration in several ways: (a) All operations are performed on variables’ original values; (b) there is no need to align cues of different scale units by assigning them weights that are difficult to interpret and to learn; and (c) relatively little computation is needed to carry out the process, which can lower operational errors that harm judgment accuracy.

Processing information sequentially is a mechanism shared by many heuristics in decision making and problem solving. In many circumstances, such heuristics can help people perform as well as or better than strategies that consider all available information simultaneously (e.g., Luan, Schooler, & Gigerenzer, 2014; Newell & Simon, 1972; Todd, Gigerenzer, & the ABC Research Group, 2012). Based on this finding and the reduced difficulty of cue integration in the process of sequential adjustment, we hypothesized that judgments following a sequential procedure will be more accurate than those following a simultaneous procedure. To test this hypothesis, we conducted four studies in which we asked both experienced and inexperienced participants for their judgments in two task domains.

## Studies 1 and 2: Judgments by professionals

### Method

#### Overview

In each study, we asked professional jewelers to estimate the price of diamonds and professional car salespeople the fuel economy of cars. The studies share the same design but were conducted in different years.

#### Task ecologies

Judgment accuracy depends on the information structure or “ecology” of a task, which can be characterized by the correlations between cues and the criterion variable, the intercue correlations, and the *R*^2^ of the best fitting regression using available cues as predictors (also known as the task’s *predictability*; Cooksey, 1996). We collected real-world data to understand the ecologies of the diamond and car tasks.

In Study 1, we obtained information on 34,251 diamonds listed at Bluenile.com in January 2009. The data set contains the price and 4Cs of each diamond. However, because only carat and color were provided to the participants, we focus on the task’s ecology involving these two cues. In the car task, we gathered information on 358 car models sold in the US market in 2009 from Yahoo.com, and the data set includes each model’s combined fuel economy, horsepower, and number of cylinders. In Study 2, we collected similar data from the same sources. This time, the data sets include 37,815 diamonds listed in June 2012 and 454 car models sold in 2012. Key statistics of the two task ecologies are reported in Table 1, showing that they differ in several important aspects.

**Table 1:** Key statistics pertaining to the task ecologies in Studies 1 and 2

Study	Environment	Bivariate Pearson correlation			Partial correlation		*R*^2^ of the best fitting regression
		Cue 1–Criterion	Cue 2–Criterion	Cue 1–Cue 2	Cue 1–Criterion	Cue 2–Criterion	
1	Diamond	+.829	+.011	−.223	+.853	+.359	.929
	Car	−.710	−.744	+.876	−.180	−.360	.715
2	Diamond	+.858	−.016	−.252	+.882	+.401	.938
	Car	−.638	−.653	+.871	−.257	−.186	.653

*Note*: The criterion, cue 1, and cue 2 are price, carat, and color in the diamond task and combined fuel economy, horsepower, and cylinders in the car task, respectively

First, whereas the two cues in the car task are similarly correlated with the criterion, one cue in the diamond task correlated with the criterion much more highly than the other. Second, the two cues in the diamond task are negatively and moderately correlated, but those in the car task are positively and highly correlated. Third, the partial correlations of the cues follow distinct patterns in the two tasks. For example, in the diamond task, the partial correlation of one cue (color) after controlling for the other (carat) is much higher than its bivariate correlation with price; such a pattern, however, does not hold in the car task. Finally, the diamond task is of substantially higher predictability than the car task.

#### Participants

Ninety-eight jewelers and car salespeople working in Singapore took part in Studies 1 and 2 (Table 2). Each participant was paid 20 Singapore dollars for their participation.

**Table 2:** Number of participants in each task and the average number of years of their professional experience

Study	Jewelers		Car salespeople	
	*N*	Average years of experience (*SD* )	*N*	Average years of experience (*SD* )
1	24	16.6 (10.1)	23	4.4 (4.2)
2	25	17.1 (9.6)	26	7.9 (4.2)

#### Design, procedure, and experimental materials

A within-subjects design with two conditions was applied in each task. In the simultaneous condition, participants were given values of two cues simultaneously and asked to provide their estimates of the criterion afterward. In the sequential condition, participants were first shown one cue – carat for the diamond task and horsepower for the car task – and asked to give an initial estimate of the criterion; after that, they were given the second cue and asked to make a second and final estimate. (Screenshots of how judgments were made in each condition can be found in the Supplementary Materials.) The order of these two conditions was counter-balanced.

An experimental session consisted of ten practice trials and two blocks of 50 trials, one for each experimental condition. In each trial, participants were given the cue values, provided their estimate, and then were shown the objective criterion value as a form of feedback. In each block, the order of trials was randomized for each participant, and the diamonds or cars in the block were a representative sample drawn from the corresponding data set, with all key statistics of the sample within ±.05 of their ecological values (Table 1).

### Results

**Fig. 2:**
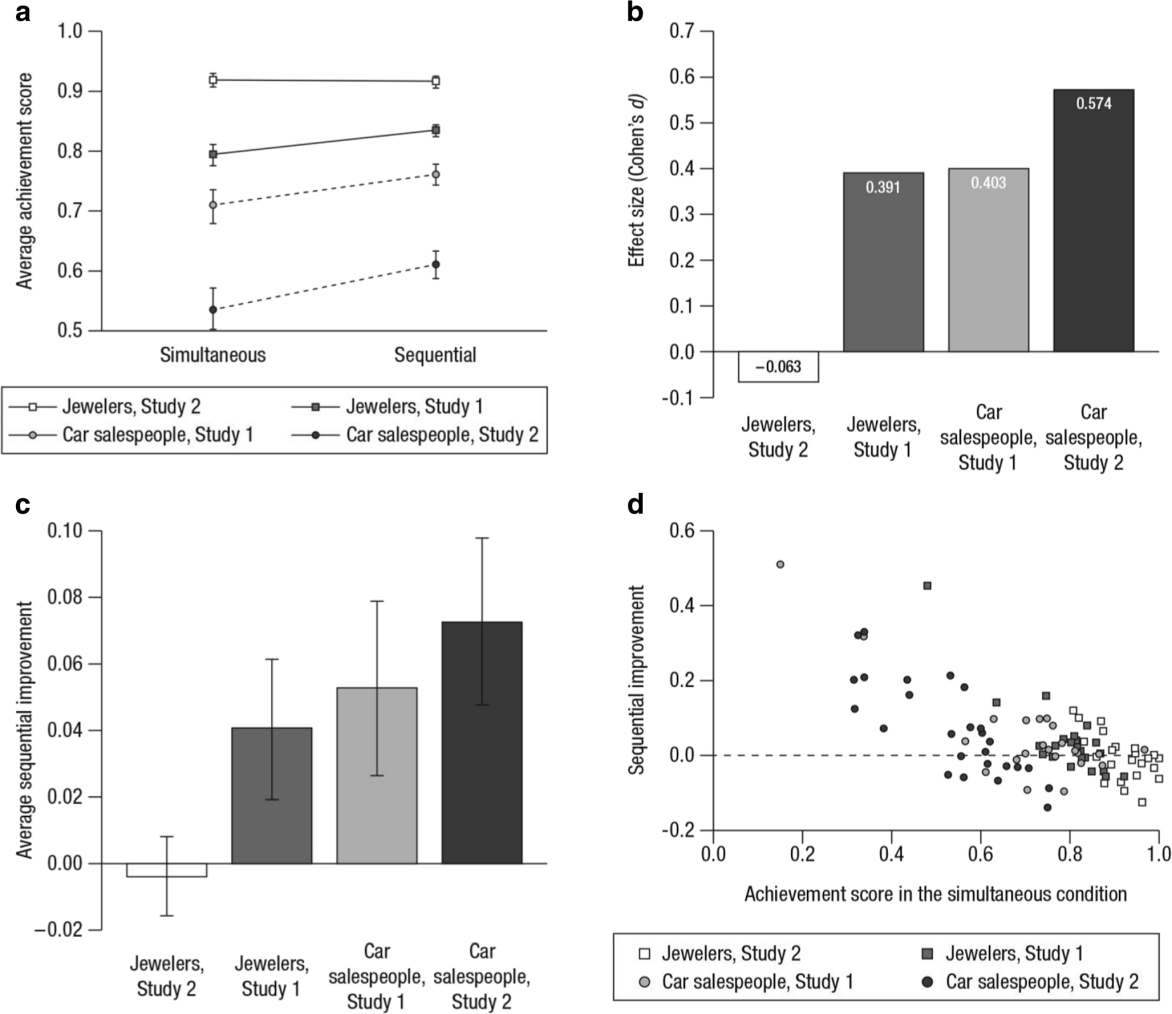
Judgments by the professionals. (**A**) The average achievement score of each participant group in each experimental condition; error bars indicate ± 1 *SE*. (**B**) The effect size, in Cohen’s *d*, of the experimental manipulation in each participant group, ordered by magnitude from left to right. (**C**) The average sequential improvement, that is, the difference between the sequential and the simultaneous conditions (former minus latter) in a participant’s achievement scores, for each participant group; error bars indicate ± 1 *SE*. (**D**) Scatterplot of participants’ sequential improvements against their achievement scores in the simultaneous condition (*N* = 98)

The most widely used measure for judgment accuracy is the “achievement score,” which is the correlation between an individual’s estimates of a criterion variable and the objective values of the variable (Cooksey, 1996). Figure 2A shows the average achievement scores of participants in each experimental group. At the aggregate level, it appears that all participant groups, except the jewelers in Study 2, judged more accurately in the sequential conditions, consistent with our hypothesis. Figure 2B shows the effect size of the experimental manipulation in each group: It was close to zero for the jewelers in Study 2 but moderately strong for the other three groups. Because of the within-subjects design, we were able to conduct an individual-level analysis on this effect. Specifically, we computed the “sequential improvement” – the difference between the two conditions in achievement score (sequential minus simultaneous) – for each participant. The average improvement of participants in each group is shown in Fig. 2C, and the results have the same pattern as those in Fig. 2B.

A ceiling effect may explain why there was no sequential improvement for the jewelers in Study 2: Because they were already quite accurate in the simultaneous condition, it is difficult to further improve their accuracy. Related to this, Figs. 2A and 2C suggest that at the aggregate level, the lower a participant group’s accuracy in the simultaneous condition, the greater their sequential improvement. Figure 2D illustrates an individual-level analysis of this relationship by plotting each participant’s sequential improvement against their achievement score in the simultaneous condition. The correlation between the two variables was negative for each participant group (all *r*s < −.597, *p*s <.002) and across all participants (*r* = −.687, *p*<.001).

These results point out an important moderating factor for our hypothesis: individuals who perform worse in the simultaneous condition tend to benefit more in the sequential condition. This finding implies that the sequential procedure would likely work for individuals with little experience in a task domain, assuming that less experience results in lower judgment accuracy in the simultaneous condition. Using college students as participants, Studies 3 and 4 tested whether this is the case.

## Studies 3 and 4: Judgments by novices

### Method

#### Participants

One hundred and forty-five students at a university in Singapore and 192 college students in China participated in Studies 3 and 4, respectively. Pre-study surveys show that participants in each study generally had little experience in judging diamond price or car fuel economy.

#### Design

In Study 3, a 2 (task) × 3 (judgment procedure) between-subjects design was applied with random participant assignment (Table 3). Besides the simultaneous condition, there were two sequential conditions in which judgments were made. In the “sequential-fixed” condition, the presentation order of the cues was fixed: The first cue was always carat in the diamond task and horsepower in the car task, the same as in the sequential condition of Studies 1 and 2. In the “sequential-optional” condition, however, participants could determine which cue they wanted to check first in each trial. This condition was added to examine how participants would search cues naturally without a pre-determined order and how this would affect their judgment accuracy.

**Table 3:** Number of participants in each experimental condition in Studies 3 and 4

Study	Task	Judgment procedure		
		Simultaneous	Sequential-fixed	Sequential-optional
3	Diamond	24	22	27
	Car	23	23	26
4	Diamond	Simultaneous	Sequential-carat first	Sequential-color first
		65	60	67

People tend to under-utilize highly valid cues when these cues are presented together with cues of little or no validity, a phenomenon known as the “dilution effect” (e.g., Nisbett, Zukier, & Lemley, 1981; Söllner, Bröder, Glöckner, & Betsch, 2014). The sequential procedure may alleviate the dilution effect, and in turn increase judgment accuracy, by isolating the highly valid cues; and because these cues are better isolated when presented first, the sequential improvement effect may be stronger in that condition. By having two sequential conditions, carat-first and color-first, Study 4 examined this hypothesis in the diamond task.^2^ The car task was not investigated, because the dilution effect should only occur when cues differ substantially in validity, which is so in the diamond but not the car task (Table 1).

#### Procedure and experimental materials

In both studies, participants completed 30 practice and 100 experimental trials. The diamonds or cars in these trials were a representative sample drawn from the corresponding task data set collected in Study 1, with all key statistics of the sample within ±.03 of their ecological values. At the start of the experiment, each participant was given a certain amount of money in their experimental account. In each trial, a monetary penalty was applied when the participant’s estimate deviated from the objective value. At the end of the trial, participants received feedback on the objective criterion value, the penalty incurred, and the money left in their account. Participants earned on average 13.15 Singapore dollars (*SD*=4.34) in Study 3 and 60.07 Chinese Yuan (*SD*=8.59) in Study 4.

### Results

**Fig. 3:**
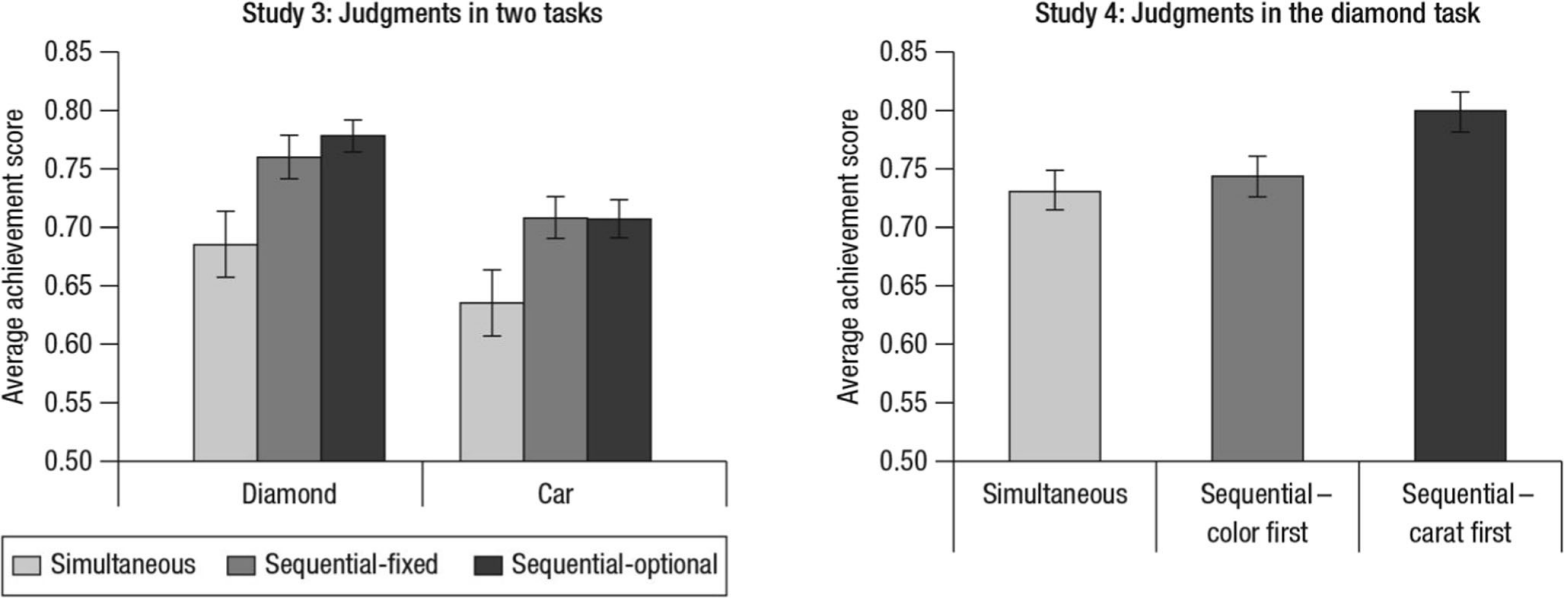
Average achievement score of participants in each experimental condition in Studies 3 and 4. The “sequential-fixed” condition in the diamond task of Study 3 was the same as the “sequential-carat first” condition of Study 4 in that carat was the first cue presented, followed by color, in both conditions. Error bars indicate ±1 *SE*

Figure 3 shows the average achievement score in each experimental condition. In Study 3, there were main effects of both task, *F*(1, 144)=11.22, *p*=.001, partial ${\eta}_p^2$ =.08, and judgment procedure, *F*(2, 143)=9.34, *p*< .001, partial ${\eta}_p^2$=.12. This indicates that, first, participants judged diamond price more accurately than car fuel economy, which was likely caused by the difference between the two tasks in predictability, and, second, accuracy depended on judgment procedure. Post-hoc Tukey tests show that participants in each sequential condition judged more accurately than those in the simultaneous condition: *p*=.003, 95% confidence interval (CI) 0.022–0.124 for sequential-fixed and *p*<.001, 95% CI 0.034–0.132 for sequential-optional; thus, a sequential procedure did improve novices’ judgment accuracy.

There was no difference between the two sequential conditions in Study 3, *p*=.887, 95% CI −0.040–0.059, suggesting that letting participants choose which cue to check first, as in the sequential-optional condition, did not reduce the sequential improvement effect. In that condition, an overwhelming majority of participants in the diamond task (24 of 27) checked the carat cue first in most trials, whereas the preference for the first cue was more evenly split among participants in the car task: 11 for horsepower and 15 for cylinders. These results match well with the cue–criterion relationships in each task (Table 1) and suggest that participants in general could learn the two cues’ relative validities and apply that knowledge in cue-order selection.

In Study 4, there was a main effect of judgment procedure, *F*(2, 190)=4.30, *p*=.015, partial ${\eta}_p^2$=.043. Post-hoc Tukey tests show that participants in the carat-first condition judged more accurately than those in the other conditions, *p*=.017, 95% CI 0.008–0.108 for simultaneous and *p*=.060, 95% CI −0.002–0.098 for color-first, and there was no difference between the latter two conditions, *p*=.872, 95% CI −0.059–0.038. Therefore, checking the much more inferior color cue first not only lowered judgment accuracy, but also made the sequential improvement disappear. These results suggest the presence of the dilution effect and are consistent with our hypothesis that checking carat first might protect participants from this detrimental effect better than the other way around. That said, the dilution effect could have only occurred in the diamond but not the car task; yet, a sequential improvement effect was still observed there.

To further understand the effect, we conducted lens model analyses of participants’ judgments in all four studies; the results can be found in the Supplementary Materials. They show that a sequential procedure improved judgment accuracy mainly by increasing the consistency of participants’ judgment policies, which was likely a result of reduced difficulty of cue integration in judgment formation.

Lastly, we compared judgment accuracy of the novices in Studies 3 and 4 with that of the professionals in Study 1, because the task ecologies in these studies were identical. We found that the novices in the sequential conditions of Study 3 performed as well as the professionals in the simultaneous condition for both the diamond task, *F*(2, 72)=0.99, *p*=.376, partial ${\eta}_p^2$= .03, and the car task, *F*(2, 71)=0.002, *p*=.998, partial ${\eta}_p^2$<001. The novices in the sequential-carat first condition of Study 4 and the jewelers in the simultaneous condition of Study 1 also performed similarly, *t*(87)=1.022, *p*=.310, Cohen’s *d*=0.116. Therefore, for people with little experience in a task domain, leading them to judge with a sequential procedure can elevate their accuracy to the level of others who have abundant experience but judge with the simultaneous procedure.

## Discussion

In four studies that covered two task domains of very different ecologies and involved both experienced and inexperienced participants, we demonstrated how a simple twist in the judgment procedure could improve judgment accuracy. Participants were given the same cue information in the simultaneous and sequential conditions; yet, most in the sequential condition achieved higher accuracy. This sequential improvement effect was less for participants who could judge more accurately in the simultaneous condition (Studies 1 and 2), but those were also the ones who had less room or need to improve. The effect even disappeared when participants were forced to process a cue of clearly low validity first (Study 4). However, when allowed to choose freely, participants rarely chose the “bad” cue as the first one and the sequential improvement effect was preserved (Study 3).

The sequential procedure increases the possibility that a sequential adjustment process is adopted in cue integration, whereas the simultaneous procedure makes weighting-and-adding a more likely choice. There have been plenty of studies on sequential adjustment (e.g., Anderson, 1981, 1996; Hogarth & Einhorn, 1992; Juslin et al., 2008), but none have investigated its effect on judgment accuracy. Drawing on decades of observations, Anderson (1981) claimed that:

In everyday life, information integration is a sequential process. Information is received a piece at a time and integrated into a continuously evolving impression. Each such impression, be it of a theoretical issue, another person, or a social organization, grows and changes over the course of time. (p. 144)

According to this statement, the simultaneous procedure that has been the standard in evaluating judgment accuracy may not fit well with the processing mode people are accustomed to using. Studies in other domains have shown that humans perform better when the problems they are asked to solve are framed in a way that make the problems match more closely those occurring in the natural environment (e.g., Cosmides, 1989; Gigerenzer & Hoffrage, 1995). Thus, the sequential procedure’s better fit to our natural judgment process may be part of the reason why it can lead to higher judgment accuracy.

There are certainly tasks where cues do not come in a clear sequence (e.g., one often sees the horsepower and cylinder information of a car in a single page) and others where they do (e.g., when seeing a diamond, its size or carat is usually the first thing that catches our attention). Regardless, our results suggest that we could still benefit from judging with a sequential procedure by *deliberately* processing cues piecemeal, and this would work particularly well in tasks we are less familiar with. With regard to cue orders, ours (Study 3) and previous studies (e.g., Katsikopoulos, Schooler, & Hertwig, 2010) have shown that people can learn to distinguish good from bad cues quickly, especially when their differences in quality are large. When the differences are small, interestingly, which cue to start with and cue orders more generally will not affect judgment accuracy much.

In 72 two-cue judgment tasks that Karelaia and Hogarth (2008) reviewed, the average achievement score was only .63 and people’s low consistency in judgment policy execution was one main reason for this result, consistent with the lens model analysis results of our data (see Supplementary Materials). There are areas of human cognition where integrating information from multiple cues can be relatively easy and/or highly accurate (e.g., Betsch & Glöckner, 2010; Cheng, Shettleworth, Huttenlocher, & Rieser, 2007). However, for novel judgment tasks where difficulties with and inefficiency in cue integration may adversely impact accuracy greatly, help is still needed.

In conclusion, our studies show the promise of a simple, step-by-step method in improving people’s judgment accuracy. This method not only enhanced the accuracy of many professionals’ judgments but also raised novices’ judgment accuracy to professional levels.

### Open Practices Statement

The data from our study are available at: https://osf.io/gzn7e (for the task ecology data sets) and https://osf.io/wbmda (for the experiment data), and none of the experiments were preregistered.

## Electronic supplementary material

ESM 1 (DOCX 173 kb)

## Footnotes

1 Diamond color ranges from “D,” the highest grade, to “Z,” the lowest, and our data only include diamonds with the top seven grades. In our regression analyses and studies, those grades were converted to numerical values, with D as 7, E as 6, and so on.

2 We thank Tilmann Betsch for suggesting the possibility of the dilution effect and the design of Study 4.
