# Supplementary material for: Improving judgment accuracy by sequential adjustment
Source: Psychon Bull Rev. 2020 Jan 2;27(1):170–7. doi: 10.3758/s13423-019-01696-5 (PMC7000513; doi:10.3758/s13423-019-01696-5)
Supplement: Supplementary file 1 — (DOCX 173 kb) [file 13423_2019_1696_MOESM1_ESM.docx]

**Supplementary Materials for**

**“Improving Judgment Accuracy by Sequential Adjustment”**

**Screenshots of How Judgments Were Made in an Experiment Trial**

Figure S1 shows a screenshot of how a judgment of diamond price was made in the simultaneous condition, and Figure S2 shows how the same kind of judgment was made in a sequential condition where carat was the first cue then color. These screenshots are from Study 3, in which participants were given a certain amount of points in their experiment account at the start of the experiment and a penalty incurred for the error in their judgment in each trial. The remaining points in their account were converted to money for payment upon the completion of the experiment. The displays in Study 4 looked the same as in Study 3, except that they were in Chinese. In Studies 1 and 2, there was no feedback information on “penalty” and “balance” after a judgment was made, because participants were paid a fixed fee for participation regardless of their judgment accuracy. Finally, in all studies and in each experimental condition, participants could see information of the previous five trials on the screen on a rolling basis. For example, if a participant was to make a judgment on trial #45, she would see the information from trial #40 to #44, highlighted in a darker background color (see Figures S1 and S2).

**Lens Model Analysis Results**

We conducted lens model analyses of participants’ judgments in all four studies. Lens model analysis is often applied to understand factors that affect judgment accuracy by breaking down the achievement score through the so-called lens model equation (Cooksey, 1996):

In the equation, *Ra* is the achievement score; *Re* is the *linear predictability*, representing how well a linear function of cues can predict the criterion variable in a certain task environment; *G* is the *matching score*, capturing how well the linear function of a person matches that of the environment; *Rs* is the *consistency score*, indicating how consistently a person executes her judgment policy; and *C* represents the part of a person’s judgments that cannot be explained by a linear function of cues. Furthermore, the product of *G* and *Rs*, *GRs*, is termed by Karelaia and Hogarth (2008) as the *linear cognitive ability*, which “quantifies the human, as opposed to the environmental, contribution to achievement and captures the extent to which judges both match task requirements and are consistent in the execution of their strategies” (p. 406).

Figure S3 shows the results of lens model analysis for participants in Studies 1 and 2. Because the linear predictability *Re* was the same for all participants in a certain task, only results of other lens model measures are shown in the figure. The indication of the results differs among the participant groups. For the car salespeople in Study 1, the sequential procedure worked by improving the consistency score *Rs*; for the car salespeople in Study 2, it worked by improving both *Rs* and the matching score *G*; for the jewelers in Study 1, the sequential procedure improved both *Rs* and *G*, but not substantially for each; and for the jewelers in Study 2, there is no difference between the two procedures in all measures. In general, these results show that when the sequential procedure improved judgment accuracy, it did so mainly by improving participants’ consistency score.

Figure S4 shows the results of lens model analysis for participants in Studies 3 and 4. A series of ANOVA tests show that in Study 3, (a) there were no effects of the two factors, task and judgment procedure, on the matching score *G*; (b) there were effects of these factors on the consistency score *Rs*; and (c) the effects were also present for linear cognitive ability *GRs*. In Study 4, the consistency score *Rs* and the linear cognitive ability *GRs* were significantly higher in the sequential-carat first condition than those in the other two conditions, suggesting that the differences in the achievement score *Ra* among the conditions was chiefly caused by their differences in *Rs*. Overall, the results from Studies 3 and 4 are consistent with those in Studies 1 and 2: The sequential improvement effect occurred mainly because a sequential procedure could improve the consistency of people’s judgment policies.

Better consistency is a critical reason why actuarial judgments derived from people’s judgments are in most cases more accurate than their own judgments (e.g., Grove, Zald, Lebow, Snitz, & Nelson, 2000). In 249 experiments analyzed in the lens model paradigm, Karelaia and Hogarth (2008) also found that both *Rs* and *GRs* are highly correlated with the achievement score *Ra*. However, despite this rather robust finding, the lens model analysis itself cannot explain the causes for high or low consistency scores.

We suspect that two factors may contribute to low consistency: the uncertainty of a judgment policy and the operational errors incurred while executing a policy. The former can be resolved by learning, while the latter may be positively related to the complexity of the policy. Because participants in each experimental condition of each our study started with similar background knowledge and went through the same number of trials, learning, and in turn policy uncertainty, should not be the factor that distinguished participants in the simultaneous and the sequential conditions. This leaves policy complexity as the likely reason for their differences in consistency. As discussed in the Introduction, the relative simplicity of the sequential adjustment process would have had helped participants in a sequential condition reduce their operational errors, leading to the higher consistency of their judgments.

*References*

Cooksey, R. W. (1996). *Judgment analysis: Theory, methods, and applications*. San Diego, CA: Academic Press.

Grove, W. M., Zald, D. H., Lebow, B. S., Snitz, B. E., & Nelson, C. (2000). Clinical versus mechanical prediction: A meta-analysis. *Psychological Assessment, 12*, 19–30.

Karelaia, N., & Hogarth, R. M. (2008). Determinants of linear judgment: A meta-analysis of lens model studies. *Psychological Bulletin, 134*, 404–426.


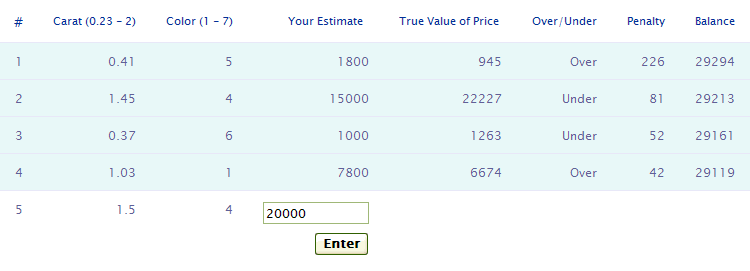


*Figure S1*. A screenshot of how a judgment of diamond price was made in a simultaneous condition.

*Figure S2*. A screenshot of how a judgment of diamond price was made in a sequential condition, in which carat was the first cue then color.

*Figure S3.* The average scores of different lens model analysis measures for participants in Studies 1 and 2. C1 = Car salespeople in Study 1; J1 = Jewelers in Study 1; C2 = Car salespeople in Study 2; J2 = Jewelers in Study 2. Error bars indicate ± 1 *SE*.

*Figure S4*. Scores of different lens model analysis measures, averaged over participants of each experimental condition, in the diamond and the car tasks in Study 3 and the diamond task of Study 4.
